# Intact leaf gas exchange provides a robust method for measuring the kinetics of stomatal conductance responses to abscisic acid and other small molecules in *Arabidopsis* and grasses

**DOI:** 10.1186/s13007-019-0423-y

**Published:** 2019-04-17

**Authors:** Paulo H. O. Ceciliato, Jingbo Zhang, Qing Liu, Xin Shen, Honghong Hu, Chen Liu, Anton R. Schäffner, Julian I. Schroeder

**Affiliations:** 1 Cell and Developmental Biology Section, Division of Biological Sciences, University of California San Diego, La Jolla, CA 92093-0116 USA; 2 National Key Laboratory of Crop Genetic Improvement, College of Life Science and Technology, Huazhong Agricultural University, Wuhan, 430070 China; 3 Biochemical Plant Pathology, Department of Environmental Sciences, Helmholtz Zentrum München, Munich, Germany

**Keywords:** Stomatal conductance, Kinetics, Guard cell physiology, Aquaporin

## Abstract

**Background:**

Guard cells perceive external and internal stimuli and regulate stomatal conductance in plants. With the use of gas exchange analyzers, time-resolved stomatal conductance responses to light intensity, [CO_2_] concentration and relative humidity changes can be measured. This is more difficult to achieve when measuring stomatal responses to small soluble molecules such as the plant hormone abscisic acid (ABA) or the bacterial peptide flagellin 22 (flg22), in particular when investigating mutants with response phenotypes.

**Results:**

A method to evaluate the dynamic effects of small molecules on stomatal conductance in a time-resolved fashion using gas exchange analyzers is presented here. ABA-induced stomatal closure was investigated by adding ABA to the transpiration stream of intact leaves placed in a microcentrifuge tube containing water. Strong ABA responses were resolved in time- and in a dose-dependent manner in wild-type *Arabidopsis* leaves, whereas the same response was not observed in leaves of the ABA-insensitive mutant *open stomata 1*-*3* (*ost1*-*3*). Moreover, when leaves of the Plasma membrane Intrinsic Protein (PIP) aquaporin quadruple mutant *pip1;1 pip1;2 pip2;1 pip2;2* were tested, robust wild-type-like responses to ABA were observed. When the bacterial peptide flg22 was added to the transpiration stream of intact wild-type leaves, a strong flg22-induced stomatal closure effect was observed. Finally, the proposed technique was further developed and optimized for evaluation of stomatal conductance responses to small molecules in leaves of grasses using the reference plant *Brachypodium distachyon*.

**Conclusions:**

Due to the variable size of stomata in *Arabidopsis* and the limited dynamic response of stomata in isolated epidermal strips, evaluation of the effect of small molecules on stomatal physiology has been challenging and has led in some cases to inconsistent results. Moreover, potential signals from the mesophyll are missing when using epidermal peels to evaluate stomatal aperture responses. Here we propose a less invasive technique which allows for time-resolved measurements of stomatal conductance responses to small molecules optimized for both *Arabidopsis* and *Brachypodium distachyon* leaves.

## Background

Plants respond to environmental change in order to maximize growth and reproduction. Stomata are specialized pores found on the surface of leaves that can both perceive and respond to external stimuli. Stomata gate CO_2_ uptake for photosynthesis and water loss through transpiration. Stomatal movements are ultimately controlled by guard cell turgor changes.

Stomatal movements can be controlled by external stimuli such as light intensity/quality [1–4], relative humidity [5–7] and CO_2_ concentration [CO_2_] changes [8, 9]. With the use of gas exchange analyzers, these parameters can be tightly controlled, and time-resolved stomatal conductance analyses have become a standard for investigating responses to these signals. On the other hand, small molecules such as the plant hormone abscisic acid (ABA) [10] and the bacterial peptide flagellin 22 (flg22) [11] can also affect stomatal movements, but quantifying kinetic responses to small molecules is more difficult as these cannot be reliably added to a gas exchange chamber. Leaf epidermal peels can be isolated and treated with these and other molecules and stomatal apertures can be measured [12]. Although this technique is broadly used, there are limitations that have to be considered, including: (a) the small and variable size of the stomata in species such as *Arabidopsis thaliana*, (b) the small area of the leaf that is evaluated by using microscopic images, (c) the invasive method of removing the epidermal peels, lacking putative important signals from the mesophyll [13, 14] and other parameters including mechanical constraints [6, 7] and (d) time-resolved measurements of the same individual stomata is labor-intensive.

Here, we propose an optimized method, which allows for time-resolved measurements of stomatal conductance and thus the kinetic response of the ensemble of hundreds of stomata in parallel to small molecules in intact leaves of both *Arabidopsis* and the grass model *Brachypodium distachyon*. The proposed technique is described here in detail and can provide a potent alternative for measuring the effect of small molecules on both the magnitude and the dynamics of stomatal movement responses.

## Methods

### Plant material and growth conditions

*Arabidopsis thaliana*, accession Columbia-0 (Col-0) and *Brachypodium distachyon* accession Bd21-3 were used as wild type references. *Arabidopsis* seeds were surface sterilized as described elsewhere [15] and cold-treated for 48 h at 4 °C. Seeds were germinated on half strength Murashige and Skoog [16, 17] basal medium supplemented with Gamborg’s vitamins (Sigma-Aldrich), 0.8% Phytoagar (Difco, Franklin Lakes, NJ, USA), 4-Morpholinoethane sulfonic acid (2.6 mM; Sigma-Aldrich) and the pH was adjusted to 5.8. Seedlings were transferred from plates to pots containing sterilized premixed soil (Sunshine Professional Blend LC1 RS; Sunshine) after 7–10 days and grown under the following conditions: 12 h light/12 h dark, 21 °C, 85–90% humidity and 90–110 µmol m^−2^ s^−1^ light. Growth of plants at this relatively high humidity was found to be helpful for investigating stimulus-induced stomatal closing in the present study.

In order to promote the growth of the leaves and the development of long and thick petioles, seedlings were kept under a transparent tray dome (7’’ height, vented humidity dome) for 2 weeks and fertilized (technigro^®^ sunshine fertilizer, 0.66 g L^−1^ of water) twice before the beginning of the experiments: first, after seedlings were transferred to pots, and second, after removing the humidity domes. Plants were ready for experiments 5–6 weeks after being transferred to pots. The *pip* quadruple mutant *pip1;1 pip1;2 pip2;1 pip2;2* was generated by crossing *pip1;1* (GK_437B11), *pip1;2* (SALK_019794), *pip2;1* (SM_3_35928), *pip2;2* (SAIL_169A03) single mutants.

For *Brachypodium*, seeds were cold-treated for 4–5 days at 4 °C, transferred to pots containing premixed soil (Sunshine Professional Blend LC1 RS; Sunshine) and kept under a tray dome for 2 weeks. Plants were fertilized once a week (technigro^®^ sunshine fertilizer, 0.66 g L^−1^ of water) and kept at the following conditions: long day (16 h light/8 h dark); 23–25 °C; 20–50% humidity, 150–250 µmol m^−2^ s^−1^ light. Plants were used 6–7 weeks after being transferred to pots.

### Leaf preparation and gas exchange

**Fig. 1:**
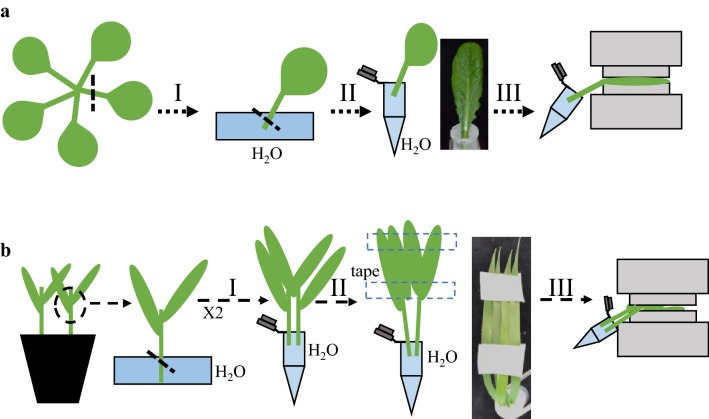
Depiction of the methods used to perform whole-leaf time-resolved ensemble analyses of small molecule responses. **a** Depiction of the approach for investigating stomatal responses to small molecules in *Arabidopsis thaliana.* **b** Depiction of the approach for investigating stomatal ABA responses in the grass *Brachypodium distachyon*. See text for details

For intact leaf gas exchange analyses using *Arabidopsis* leaves, petioles are first cut using a new razor blade and the cut surface of the petioles was immediately transferred to a petri dish filled and submerged in milli-Q water (Fig. 1aI). Petioles are cut a second time under water using a razor blade. The second cut under water is a crucial step of the proposed technique and can be difficult to make. For the second cut it is recommended: (a) approximately one-third of the petiole should be cut but no more. Longer petioles are better for the following steps; (b) the razor blade should be positioned perpendicular to the petiole and at an oblique angle; (c) the cut should be made by gently moving the razor blade back and forth and not by pressing the blade against the petiole and (d) a microcentrifuge tube filled with milli-Q water and closed with plastic paraffin film (Parafilm M) should be prepared in advance. After the parafilm is placed on the tube, two holes are made using fine tweezers, one for the petiole and the second for adding the treatment to the water. After the second cut is made the petiole, with a droplet of water on its cut end, is immediately transferred to the microcentrifuge tube filled with milli-Q water (Fig. 1a, II). The droplet on the end of the petiole is essential to avoid xylem embolism. Leaves are placed inside the gas exchange chamber (Fig. 1a, III) and equilibrated for 45–90 min to reach a stable stomatal conductance before the beginning of the experiments. Experiments were started at least 1 h after growth chamber light onset.

For intact leaf gas exchange in *Brachypodium*, the petioles are first cut 1.5–2.0 cm below the junction between two leaves using a razor blade and immediately placed in a petri dish filled with milli-Q water (Fig. 1b, I). The second cut is made under water using a razor blade positioned perpendicular to the petiole and in an oblique angle and the petiole is transferred to a microcentrifuge tube filled with milli-Q water and closed with parafilm. A second pair of leaves is prepared as described above and added together to the first pair in the same hole in the microcentrifuge tube (Fig. 1b, II). The leaves are taped together using surgical tape (micropore 3 M), leaving the middle area of the leaves free for placement into the gas exchange chamber (Fig. 1b, III), where they equilibrate for 2 h before the beginning of the experiments.

Stomatal conductance to water (gsw) was measured using a portable gas exchange system (LI6400 and LI-6800, LI-COR, Lincoln, Nebraska) with the following conditions: the LED light source was set at 150 μmol m^−2^ s^−1^ (10% blue light), gas exchange temperature was 21 °C, relative humidity in the chamber was kept between 74 and 78% as high relative humidity is very important in these experiments for analyzing petiole-fed small molecules-induced stomatal closing. Airflow was set to 200 revolutions per minute (rpm) and the CO_2_ concentration at 400 ppm. After allowing the stomatal conductance to settle at a steady state level, steady-state stomatal conductance was recorded for 10 min prior to the addition of the treatment at the indicated concentrations. The data represent N ≥ 3 leaves per genotype per treatment.

### RT-PCR analysis of *pip* mutants

Total RNAs from 4-week-old plants were extracted using the TRIzol Reagent (Invitrogen, Carls-bad, CA, USA) or the innuPREP Plant RNA kit (Analytik Jena, Jena, Germany) and reverse transcribed using the M-MLV reverse transcriptase (Promega or Qiagen, Hilden, Germany) according to the manufacturer’s instructions. The presence of functional transcripts was assessed by RT-PCR using primer pairs flanking the insertion site of each mutant (**PIP1;1F**- CAGAGCTTTACAATTTCTCTCTACA, **PIP1;1R**- CACAGTGTTAGCTCCTCCTCCT; **PIP1;2F**- CTGGTTTCTCCGATCTAACGA, **PIP1;2R**- GCATTTTGATCCGATGTTACAA; **PIP2;1F**- AACATATAACGTTGGCAAAAA, **PIP2;1R**- TGGTTAAGACAGGGTTAGTCA; **PIP2;2F**- AAGTTATAGAAATGGCCAAAGAC, **PIP2;2R**- CTCAAACGTTGGCTGCACTTCTG). Expression levels of the housekeeping gene *TUB9* was used as control for cDNA synthesis (**TUB9F-** GTACCTTGAAGCTTGCTAATCCTA, **TUB9R-** GTTCTGGACGTTCATCATCTGTTC).

### Stomatal aperture measurements

Three-week-old *Arabidopsis* plants were grown in a growth chamber at 21 °C at 70% humidity, 16 h light/8 h dark regime with a white light intensity 80 µmol m^−2^ s^−1^. Leaf epidermal layers were pre-incubated in opening buffer (10 mM MES, 10 mM KCl and 10 mM CaCl_2_ at pH 6.15) for 2 h in a growth chamber with a light intensity of 150 µmol m^−2^ s^−1^ at 21 °C and then incubated with buffers supplemented with 10 μM ABA for 1 h. Stomata were tracked, and apertures were measured using ImageJ. Stomatal assays were conducted as genotype-blinded.

## Results

### Intact leaf gas exchange enables time-resolved evaluation of ABA effects on stomatal movements

**Fig. 2:**
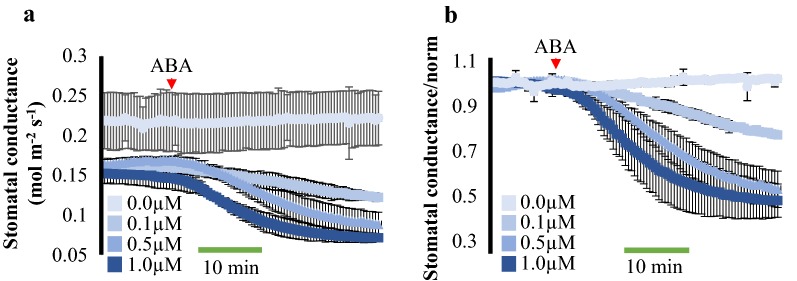
Time-resolved stomatal conductance analysis of ABA responses in intact *Arabidopsis* leaves. Time-resolved stomatal conductance responses at the indicated ABA concentrations in wild-type (Col-0) were analyzed using whole-leaf gas exchange analyses. **a** Stomatal conductance in mol m^−2^ s^−1^. **b** Data shown in **a** were normalized to the average of the first 10 min of stomatal conductance values recorded. ABA was added to the transpiration stream (red arrowhead). N = 3 leaves per condition ± SD

To monitor abscisic acid (ABA) responses in intact leaves, five-week-old leaves from wildtype (Col-0) plants were excised and the petioles were immediately submerged into water. Then, their petioles were cut a second time under water and placed in a microcentrifuge tube with the petiole tips submerged in water. Leaves were clamped in a gas exchange chamber for time-resolved measurements of stomatal conductance (Fig. 1a). Leaves were equilibrated for 70 min inside the gas exchange chamber, before the addition of mock treatment or abscisic acid (ABA). ABA was applied to the water in the microcentrifuge tube in three final concentrations: 0.1, 0.5 and 1.0 µM. For all three concentrations tested, a decrease in stomatal conductance was observed within 5–10 min of ABA addition (Fig. 2a, b). The slope of the stomatal conductance change (from 8 to 15 min after the addition of ABA) was calculated for all treatments and compared to mock treatments. All three ABA concentrations tested showed significant changes in the negative slope of the stomatal conductance when compared to the ethanol mock treatment (Slope of mock treatments: average = 0.0002 mol m^−2^ s^−1^ min^−1^ ± 0.001, slope of 0.1 μM ABA treatment: average = − 0.0005 mol m^−2^ s^−1^ min^−1^ ± 0.0001, slope of 0.5 μM ABA treatment: average = − 0.0016 mol m^−2^ s^−1^ min^−1^ ± 0.0004 and slope of 1.0 μM ABA treatment: average = − 0.0020 mol m^−2^ s^−1^ min^−1^ ± 0.0008. One-Way ANOVA, *p* value ≤ 0.05). Moreover, a more rapid decrease in stomatal conductance was consistently observed for 0.5 and 1.0 µM of ABA when compared to the 0.1 µM ABA treatment. Note that steady-state stomatal conductance values for the mock treatment in Fig. 2 are slightly higher than the values observed in ABA-treated leaves. This variation in steady-state stomatal conductance is often observed between plants grown at separate batches as done here (mock) [18].

**Fig. 3:**
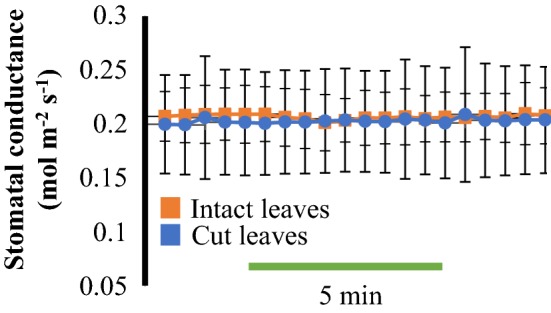
Time-resolved stomatal conductance analysis of the same leaves before and after the cut of the petioles. The same three leaves from three plants were evaluated before (in intact plants) and after the cut of their petioles. Time resolved steady-state stomatal conductance was measured after 70 min of insertion of the leaves into a gas exchange analyzer chamber. N = 3 leaves per condition ± SD

To test whether steady-state stomatal conductance levels were comparable between intact and cut leaves, the same three independent leaves from three plants were evaluated before and after cutting their petioles. Leaves were first equilibrated inside the gas exchange chamber for 70 min and stomatal conductance was recorded for 10 min in intact plants. The same leaves were then cut as described above and inserted into the gas exchange chamber to again equilibrate for 70 min. Steady-state stomatal conductance of cut leaves was recorded for 10 min after equilibration in the gas exchange chamber and were very similar to steady-state stomatal conductance values of intact leaves (Fig. 3).

**Fig. 4:**
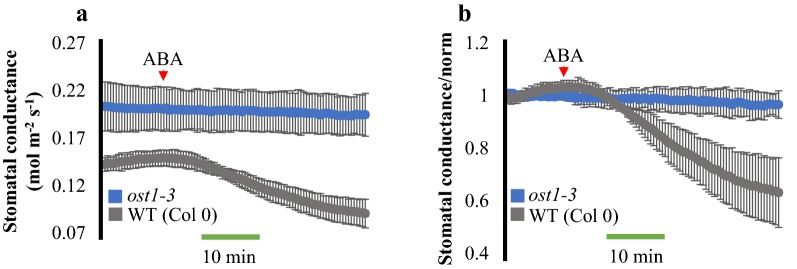
*ost1*-*3* mutant is insensitive to ABA in time-resolved stomatal conductance analysis. Time-resolved stomatal conductance responses in response to ABA (2 µM) in wild-type (Col-0) and *ost1*-*3* (Col-0 ecotype) mutant were analyzed using a whole-leaf gas exchange analyzer. **a** Stomatal conductance in mol m^−2^ s^−1^. **b** Data shown in **a** were normalized to the average of the first 10 min of stomatal conductance values recorded. ABA was added to the transpiration stream (red arrowhead). N = 3 leaves per genotype ± SD

The protein kinase “OPEN STOMATA1” (OST1) is a key component of ABA signal transduction in guard cells and the *ost1*-*3* mutant is strongly impaired in ABA-induced stomatal closure [19, 20]. To measure ABA responses in intact leaves of the *ost1*-*3* mutant, ABA was added to the microcentrifuge tubes containing WT (Col-0) or *ost1*-*3* leaves at a final concentration of 2 µM. Stomatal conductance in *ost1*-*3* leaves was not strongly affected by the addition of ABA, whereas the same treatment in parallel-grown WT (Col-0) leaves strongly reduced stomatal conductance (Fig. 4a). The steady-state stomatal conductance of *ost1*-*3* mutants before the treatment with ABA was higher than the steady-state stomatal conductance in WT under the imposed conditions from parallel grown plants (Fig. 4a). To better compare and visualize the effect of ABA on stomatal conductance, the data were normalized to the average stomatal conductance values during the first 10 min, before the addition of ABA (Fig. 4b). Together, these data suggest that the measurable decrease in stomatal conductance by the addition of ABA to the transpiration stream of intact leaves is mediated by ABA and this protocol enables analyses of the time-resolved ensemble average of hundreds of stomatal apertures in intact leaves in each experiment.

### Aquaporin *pip1;1 pip1;2 pip2;1 pip2;2* quadruple mutant leaves show a WT-like response to ABA in intact leaves

Aquaporins are physiologically relevant water channels that facilitate the passage of water across cell membranes. As guard cells use cell turgor to regulate and fine-tune stomatal apertures, guard cell-expressed aquaporins play an important role in this process by facilitating water diffusion [21–25]. However, the large gene family of the plasma membrane intrinsic protein (PIP) aquaporins [26] have rendered it difficult to investigate the role of plasma membrane aquaporins in ABA-induced stomatal closing. Moreover, whether single mutant alleles in the *pip2;1* gene impair ABA-induced stomatal closing remains a matter of debate [27, 28].

**Fig. 5:**
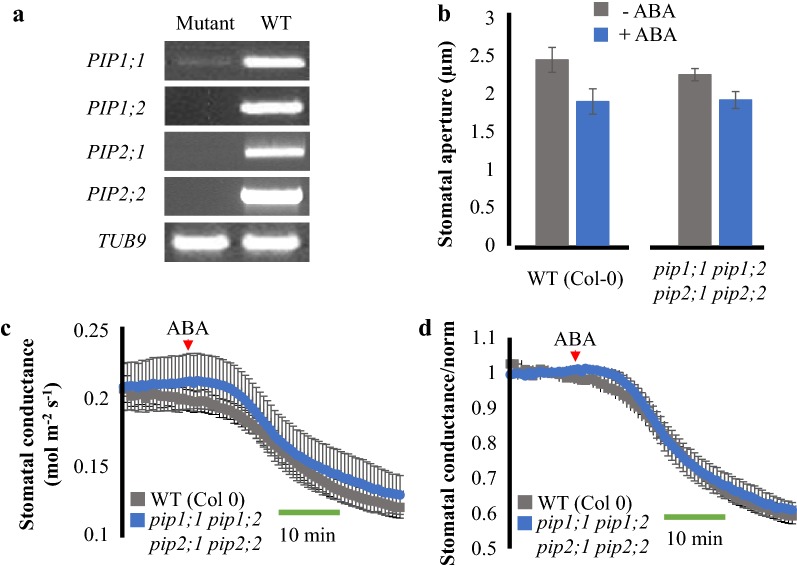
Aquaporin *pip* quadruple mutant is responsive to ABA. **a** Expression of *PIP* genes in WT (Col 0) and *pip1;1, pip1;2, pip2;1 and pip2;2* mutants. Expression of tubulin 9 is shown as control. **b** Analyses of stomatal movements in response to ABA. Leaf epidermal layers were incubated with ± ABA (10 μM ABA) and the stomatal aperture was scored (N = 10 stomata per genotype per treatment). **c** Stomatal conductance of detached leaves from wild type (Col-0) (n = 3) and *pip1;1/pip1;2/pip2;1/pip2;2* quadruple mutant (n = 4) plants in response to 2 μM ABA. **d** Normalized data shown in panel **c**. Data shown in **c** were normalized to the average of the first 10 min of stomatal conductance values recorded. ABA was added to the transpiration stream (red arrowhead). N > 3 leaves ± SEM per genotype

We therefore generated a *pip* quadruple mutant (*pip1;1 pip1;2 pip2;1 pip2;2*) using the same *pip2;1*-*2* mutant allele used in previous research [27, 28] to test whether higher order *pip* mutant leaves have impaired responses to ABA in intact leaves. RT-PCR analyses did not detect transcripts of *PIP1;2,* *PIP2;1* and *PIP 2;2*, whereas the detection of *PIP1;1* was substantially lower in the single mutants used to generate the quadruple mutant, when compared to WT (Fig. 5a). In stomatal aperture measurements using isolated epidermal strips, little difference was noted in response to ABA (10 µM) between *pip* quadruple mutant and WT leaves (One-Way ANOVA, *p* value > 0.05) (Fig. 5b). However, the stomatal response in stomatal aperture experiments was quite small. In intact leaf gas exchange analyses, the steady-state stomatal conductance of *pip* quadruple mutant leaves was similar to WT leaves under the imposed conditions (Fig. 5c). When ABA (2 µM) was applied to the transpiration stream of intact *pip* quadruple mutant leaves, a WT-like decrease in stomatal conductance was observed (Fig. 5c, d). Our results show that intact quadruple mutant leaves of these *pip* aquaporins are highly responsive to ABA treatments in stomatal conductance and indistinguishable from the wild type response in intact leaves both in terms of the time-dependent kinetics of the response and the magnitude of the ABA response.

### The defense elicitor peptide flagellin 22 leads to a rapid decrease in stomatal conductance when applied to the transpiration stream of *Arabidopsis* leaves

Microbe-associated molecular patterns (MAMPs) are molecules recognized by plants during pathogen infection that trigger several defense-related responses, which includes stomatal closure. The bacterial peptide flagellin 22 (flg22) is recognized by the FLAGELLIN SENSITIVE2 (FLS2) receptor kinase [29] and has been reported to lead to a stomatal closure response [30], when nano-infused in *Arabidopsis* leaves [11].

**Fig. 6:**
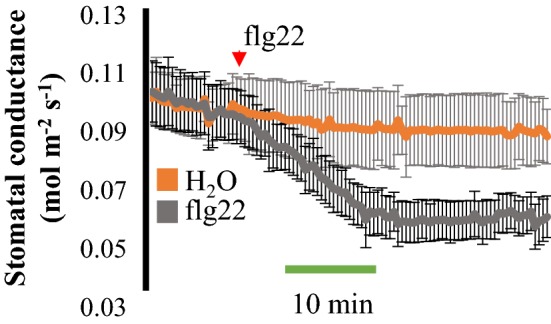
Time-resolved analysis of stomatal responses to the defense elicitor peptide flg22 in intact leaves. Time-resolved stomatal conductance responses at a final concentration of 10 µM flagellin 22 (flg22) in wild-type (Col-0) was analyzed in intact leaves using a whole-leaf gas exchange analyzer. N = 3 leaves per condition ± SD

To test whether the flg22 peptide could affect stomatal conductance in intact leaves, we applied 10 µM flg22 or mock treatment to the transpiration stream of *Arabidopsis* WT (Col-0) leaves. Flg22 peptide treatments clearly decreased stomatal conductance of intact leaves within 20 min, compared to mock treatment controls (Fig. 6). Our data confirm that the flg22 peptide is a strong regulator of stomatal closure and can be effective when applied to the leaf transpiration stream. These data suggest that the present method is suitable for evaluating the kinetic response of diverse molecules on stomatal conductance regulation in intact leaves.

### Optimization of intact leaf gas exchange in grasses using *Brachypodium distachyon*

Understanding the dynamics of stomatal movements in response to defined stimuli is relevant in many plant species, including in the grasses that have unique dumbbell-shaped stomata [31]. Although research on the reference plant, *Arabidopsis thaliana*, is crucial for developing knowledge of stomatal response mechanisms in dicotyledon plants, research is necessary to further our knowledge on stomatal responses in grasses. Recently, in the emerging model organism *Brachypodium distachyon* [32], it was observed that stomatal subsidiary cells play a key role in enhancing stomatal responsiveness [33], which can be best observed in time-resolved experiments.

**Fig. 7:**
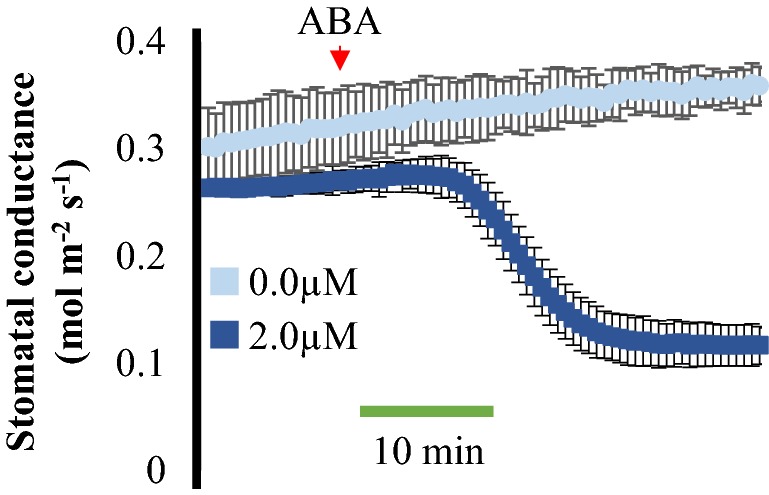
Time-resolved analysis of stomatal conductance response to ABA in intact leaves of *Brachypodium distachyon.* Time-resolved stomatal conductance responses at the imposed ABA concentrations (0 and 2 µM) in wild-type Bd21-3 Brachypodium plants were analyzed using whole-leaf gas exchange. N = 3 experiments, 4 leaves in each experiment ± SD

In order to measure ABA-dependent stomatal closing in *Brachypodium* leaves, our protocol was adapted and optimized (Fig. 1b). ABA or mock ethanol treatment were added to the microfuge tube and stomatal conductance was recorded. Approximately 5 min after the addition of ABA, a rapid decrease in stomatal conductance was observed and a new plateau was rapidly reached within 15 min (Fig. 7). Our results suggest that this method for time-resolved kinetic evaluation of small molecules effects on stomatal conductance in intact leaves can be applied to different species with distinct leaf sizes and morphologies.

## Discussion

Stomatal responses to external and internal stimuli are important for plant survival and quantifying these responses can be difficult. With the use of gas exchange analyzers, responses to a diverse range of stimuli, from light quality and light intensity to [CO_2_] concentration changes, can be automatically controlled and stomatal conductance responses can be measured in time.

On the other hand, small molecules such as the plant hormone ABA and the bacterial peptide flg22 have major impact on stomatal physiology [11, 34]. However, to date there has been only limited use of automated techniques to robustly measure these responses. Here we provide detailed information for a simple method in which leaf petioles are placed inside a centrifuge tube containing water and leaves are clamped into a gas exchange chamber (Fig. 1) for evaluation of stomatal conductance responses to small soluble molecules. With this technique, stomatal responses to ABA were measured in time and showed dose-dependency (Fig. 2a, b). The steady-state stomatal conductance of leaves was measured before and after the cuts of the petiole and showed similar values (Fig. 3), suggesting that the cut of the petioles pose little effect on stomatal conductance when following the described protocol. In addition, the petioles could be submerged in water for the first cut to avoid any xylem embolism and to possibly facilitate steady-state stomatal conductance recovery. ABA-dependent decrease in stomatal conductance was not observed when leaves from the *ost1*-*3* mutant were treated with ABA (Fig. 4).

To evaluate the contribution of four abundant plasma membrane aquaporins, we tested a *pip* (*pip1;1 pip1;2 pip2;1 pip2;2*) quadruple mutant’s response to ABA in both epidermal peels and intact leaves. The expression of all four *PIP* genes affected in our mutants were evaluated using RT-PCR. This analysis confirmed the loss-of-function mutation of *PIP1;2*, *PIP2;1* and *PIP2;2*, while a low expression of the *PIP1;1* gene could still be detected in the *pip1;1* mutant, probably through a partially correct splicing of the T-DNA insertion within an intron allele (Fig. 5a). While stomatal aperture data indicate a possible, but non-significant, reduction in ABA-responsiveness (Fig. 5b), in intact leaf gas exchange experiments the *pip* quadruple mutant leaves show WT-like responses to ABA (Fig. 5c, d). The present data suggest that time-resolved measurements are more adequate for comparing genotypes due to the large dynamic range of the intact leaf response. These findings lie in contrast to a study that suggested dramatic impairment in ABA-induced stomatal closing in leaf epidermal peels of the same single *pip2;1* mutant allele [27]. In contrast, another study showed intact ABA responses in the single *pip2;1* mutant [28], consistent with the present study. These data point to the hypothesis that even higher order *pip* mutants would be required to observe any noticeable phenotype in the ABA response when studying PIP contribution in a whole leaf context.

The bacterial peptide flg22 was reported to reproducibly induce stomatal closure in a SLAC1-dependent manner when flg22 was nano-infused in stomata [11]. Although this elegant technique clearly confirmed the effect of flg22 on stomatal closure, the effect of this peptide had not yet been investigated in stomatal conductance of intact leaves. When applied to the transpiration stream of intact leaves, flg22 peptide rapidly induced a decrease in stomatal conductance (Fig. 6). These data suggest that small peptides can travel through the xylem of leaves and reach guard cells, regulating stomatal aperture and gas exchange in intact leaves.

Similar techniques have been recently used to measure ABA-dependent stomatal closure in *Arabidopsis* [35–38] and also in several other plant species such as *Phoenix dactylifera* [39], *Vicia faba* [40], tomato (*Solanum lycopersicum*) [41] and barley (*Hordeum vulgare*) [42]. In the present study we provide a detailed protocol for robust small molecule response analyses. In the case of grasses, the stomata are surrounded by subsidiary cells which respond to external and internal stimuli with changes in turgor pressure that are inverted with respect to the guard cell response. Furthermore, the subsidiary cells have been shown to maximize stomatal responses [33]. Measuring the effect of small molecules on stomatal conductance in grasses will be important towards understanding stomatal physiology in monocots. Our technique was optimized and rapid ABA-mediated decreases in stomatal conductance were resolved in intact leaves of the reference grass species *Brachypodium distachyon* (Fig. 7). This allowed us to quantify not only the final magnitude of ABA responses, but importantly, the time course of the ensemble average of stomata in intact leaves could be resolved.

## Conclusions

Understanding how guard cells respond to stresses and signals is a subject of interest in plant biology and numerous advances have been made. Signals that modulate stomatal movements and that can be controlled by gas exchange analyzers, such as light and CO_2_ concentration, have been extensively investigated. It has become a standard in this field to include time-resolved data on stomatal conductance for stimuli that can be automatically adjusted. Here we present a detailed method applying small soluble molecules that has a substantially larger dynamic range than epidermal peel experiments and enables kinetic analyses. We recommend this approach for evaluation of stomatal conductance responses to small molecules, particularly in mutants which have intermediate phenotypes. The protocol is described in detail here and optimized for two key model organisms, *Arabidopsis* and the grass *Brachypodium distachyon*.

## References

[1] Kinoshita T, Shimazaki K-I (1999). Blue light activates the plasma membrane H+-ATPase by phosphorylation of the C-terminus in stomatal guard cells. EMBO J..

[2] Kinoshita T, Doi M, Suetsugu N, Kagawa T, Wada M, Shimazaki K-I (2001). phot1 and phot2 mediate blue light regulation of stomatal opening. Nature.

[3] Inoue S-I, Kinoshita T, Matsumoto M, Nakayama KI, Doi M, Shimazaki K-I (2008). Blue light-induced autophosphorylation of phototropin is a primary step for signaling. Proc Natl Acad Sci USA.

[4] Assmann SM (1993). Signal transduction in guard cells. Ann Rev Cell Biol.

[5] Bauer H, Ache P, Lautner S, Fromm J, Hartung W, Al-Rasheid KA (2013). The stomatal response to reduced relative humidity requires guard cell-autonomous ABA synthesis. Curr Biol CB.

[6] Kollist H, Zandalinas SI, Sengupta S, Nuhkat M, Kangasjärvi J, Mittler R (2018). Rapid responses to abiotic stress: priming the landscape for the signal transduction network. Trends Plant Sci.

[7] Merilo E, Yarmolinsky D, Jalakas P, Parik H, Tulva I, Rasulov B (2018). Stomatal VPD response: there is more to the story than ABA. Plant Physiol.

[8] Young JJ, Mehta S, Israelsson M, Godoski J, Grill E, Schroeder JI (2006). CO_2_ signaling in guard cells: calcium sensitivity response modulation, a Ca^2+^-independent phase, and CO_2_ insensitivity of the *gca2* mutant. Proc Natl Acad Sci USA.

[9] Engineer CB, Hashimoto-Sugimoto M, Negi J, Israelsson-Nordstrom M, Azoulay-Shemer T, Rappel W-J (2016). CO_2_ sensing and CO_2_ regulation of stomatal conductance: advances and open questions. Trends Plant Sci.

[10] Weiner JJ, Peterson FC, Volkman BF, Cutler SR (2010). Structural and functional insights into core ABA signaling. Curr Opin Plant Biol.

[11] Deger AG, Scherzer S, Nuhkat M, Kedzierska J, Kollist H, Brosché M (2015). Guard cell SLAC1-type anion channels mediate flagellin-induced stomatal closure. N Phytol.

[12] Weyers JDB, Travis AJ (1981). Selection and preparation of leaf epidermis for experiments on stomatal physiology. J Exp Bot.

[13] Mott KA, Sibbernsen ED, Shope JC (2008). The role of the mesophyll in stomatal responses to light and CO_2_. Plant, Cell Environ.

[14] Lawson T, Simkin AJ, Kelly G, Granot D (2014). Mesophyll photosynthesis and guard cell metabolism impacts on stomatal behaviour. N. Phytol.

[15] Lindsey BE, Rivero L, Calhoun CS, Grotewold E, Brkljacic J (2017). Standardized method for high-throughput sterilization of arabidopsis seeds. J Vis Exp JoVE.

[16] Murashige T, Scholar FSG (1962). A revised medium for rapid growth and bioassay with tobacco tissue culture. Phisiol Plant.

[17] Gamborg OL, Miller RA, Ojima K (1968). Nutrient requirements of suspension cultures of soybean root cells. Exp Cell Res.

[18] Azoulay-Shemer T, Bagheri A, Wang C, Palomares A, Stephan AB, Kunz H-H (2016). Starch biosynthesis in guard cells but not in mesophyll cells is involved in CO_2_-induced stomatal closing. Plant Physiol.

[19] Mustilli A-C, Merlot S, Vavasseur A, Fenzi F, Giraudat J (2002). Arabidopsis OST1 protein kinase mediates the regulation of stomatal aperture by abscisic acid and acts upstream of reactive oxygen species production. Plant Cell.

[20] Yoshida R, Hobo T, Ichimura K, Mizoguchi T, Takahashi F, Aronso J (2002). ABA-activated SnRK2 protein kinase is required for dehydration stress signaling in arabidopsis. Plant Cell Physiol.

[21] Uehlein N, Lovisolo C, Siefritz F, Kaldenhoff R (2003). The tobacco aquaporin NtAQP1 is a membrane CO_2_ pore with physiological functions. Nature.

[22] Maurel C, Verdoucq L, Luu D-T, Santoni V (2008). Plant aquaporins: membrane channels with multiple integrated functions. Annu Rev Plant Biol.

[23] Kaldenhoff R, Ribas-Carbo M, Sans JF, Lovisolo C, Heckwolf M, Uehlein N (2008). Aquaporins and plant water balance. Plant, Cell Environ.

[24] Heinen RB, Bienert GP, Cohen D, Chevalier AS, Uehlein N, Hachez C (2014). Expression and characterization of plasma membrane aquaporins in stomatal complexes of Zea mays. Plant Mol Biol.

[25] Chaumont F, Tyerman SD (2014). Aquaporins: highly regulated channels controlling plant water relations. Plant Physiol.

[26] Quigley F, Rosenberg JM, Shachar-Hill Y, Bohnert HJ. From genome to function: the Arabidopsis aquaporins. Genome Biol 2002;3:RESEARCH0001. 10.1186/gb-2001-3-1-research0001 PMC150448 11806824

[27] Grondin A, Rodrigues O, Verdoucq L, Merlot S, Leonhardt N, Maurel C. Aquaporins contribute to ABA-triggered stomatal closure through OST1-mediated phosphorylation. Plant Cell 2015;27:tpc.15.00421–1954. 10.1105/tpc.15.00421. 10.1105/tpc.15.00421 PMC4531361 26163575

[28] Wang C, Hu H, Qin X, Zeise B, Xu D, Rappel W-J (2016). Reconstitution of CO_2_ regulation of SLAC1 anion channel and function of CO_2_-permeable PIP2;1 aquaporin as carbonic ANHYDRASE4 interactor. Plant Cell.

[29] Gómez-Gómez L, Boller T (2000). FLS2: an LRR receptor–like kinase involved in the perception of the bacterial elicitor flagellin in arabidopsis. Mol Cell.

[30] Melotto M, Underwood W, Koczan J, Nomura K, He SY (2006). Plant stomata function in innate immunity against bacterial invasion. Cell.

[31] McKown KH, Bergmann DC (2018). Grass stomata. Curr Biol CB.

[32] Raissig MT, Abrash E, Bettadapur A, Vogel JP, Bergmann DC (2016). Grasses use an alternatively wired bHLH transcription factor network to establish stomatal identity. Proc Natl Acad Sci USA.

[33] Raissig MT, Matos JL, Gil MXA, Kornfeld A, Bettadapur A, Abrash E (2017). Mobile MUTE specifies subsidiary cells to build physiologically improved grass stomata. Science.

[34] Kim T-H, Böhmer M, Hu H, Nishimura N, Schroeder JI (2010). Guard cell signal transduction network: advances in understanding abscisic acid, CO_2_, and Ca^2+^ signaling. Annu Rev Plant Biol.

[35] Batool S, Uslu VV, Rajab H, Ahmad N, Waadt R, Geiger D (2018). Sulfate is incorporated into cysteine to trigger ABA production and stomatal closure. Plant Cell.

[36] Zhang J, Wang N, Miao Y, Hauser F, McCammon JA, Rappel W-J (2018). Identification of SLAC1 anion channel residues required for CO_2_/bicarbonate sensing and regulation of stomatal movements. Proc Natl Acad Sci USA.

[37] Hauser F, Ceciliato PHO, Lin Y-C, Guo D, Gregerson JD, Abbasi N (2018). A seed resource for screening functionally redundant genes and isolation of new mutants impaired in CO_2_ and ABA responses. J Exp Bot.

[38] Park J, Kim T-H, Takahashi Y, Schwab R, Dressano K, Stephan AB (2019). Chemical genetic identification of a lectin receptor kinase that transduces immune responses and interferes with abscisic acid signaling. Plant J Cell Mol Biol.

[39] Müller HM, Schäfer N, Bauer H, Geiger D, Lautner S, Fromm J (2017). The desert plant Phoenix dactylifera closes stomata via nitrate-regulated SLAC1 anion channel. N Phytol.

[40] Felle HH, Hanstein S, Steinmeyer R, Hedrich R (2000). Dynamics of ionic activities in the apoplast of the sub-stomatal cavity of intact Vicia faba leaves during stomatal closure evoked by ABA and darkness. Plant J Cell Mol Biol.

[41] Wilkinson S, Davies WJ (2008). Manipulation of the apoplastic pH of intact plants mimics stomatal and growth responses to water availability and microclimatic variation. J Exp Bot.

[42] Schäfer N, Maierhofer T, Herrmann J (2018). A tandem amino acid residue motif in guard cell SLAC1 anion channel of grasses allows for the control of stomatal aperture by nitrate. Curr Biol.

